# Genetic pathogenesis of immunoglobulin light chain amyloidosis: basic characteristics and clinical applications

**DOI:** 10.1186/s40164-021-00236-z

**Published:** 2021-07-20

**Authors:** Linchun Xu, Yongzhong Su

**Affiliations:** 1 Shantou University Medical College, Shantou, 515031 Guangdong China; 2 The First Affiliated Hospital of Shantou University Medical College, Shantou, 515041 Guangdong China; 3 Department of Hematology, The First Affiliated Hospital of Shantou University Medical College, Shantou, 515041 Guangdong China

**Keywords:** Immunoglobulin light chain amyloidosis, Genetic aberrations, Prognosis, Therapy

## Abstract

Immunoglobulin light chain amyloidosis (AL) is an indolent plasma cell disorder characterized by free immunoglobulin light chain (FLC) misfolding and amyloid fibril deposition. The cytogenetic pattern of AL shows profound similarity with that of other plasma cell disorders but harbors distinct features. AL can be classified into two primary subtypes: non-hyperdiploidy and hyperdiploidy. Non-hyperdiploidy usually involves immunoglobulin heavy chain translocations, and t(11;14) is the hallmark of this disease. T(11;14) is associated with low plasma cell count but high FLC level and displays distinct response outcomes to different treatment modalities. Hyperdiploidy is associated with plasmacytosis and subclone formation, and it generally confers a neutral or inferior prognostic outcome. Other chromosome abnormalities and driver gene mutations are considered as secondary cytogenetic aberrations that occur during disease evolution. These genetic aberrations contribute to the proliferation of plasma cells, which secrete excess FLC for amyloid deposition. Other genetic factors, such as specific usage of immunoglobulin light chain germline genes and light chain somatic mutations, also play an essential role in amyloid fibril deposition in AL. This paper will propose a framework of AL classification based on genetic aberrations and discuss the amyloid formation of AL from a genetic aspect.

## Background

**Fig. 1:**
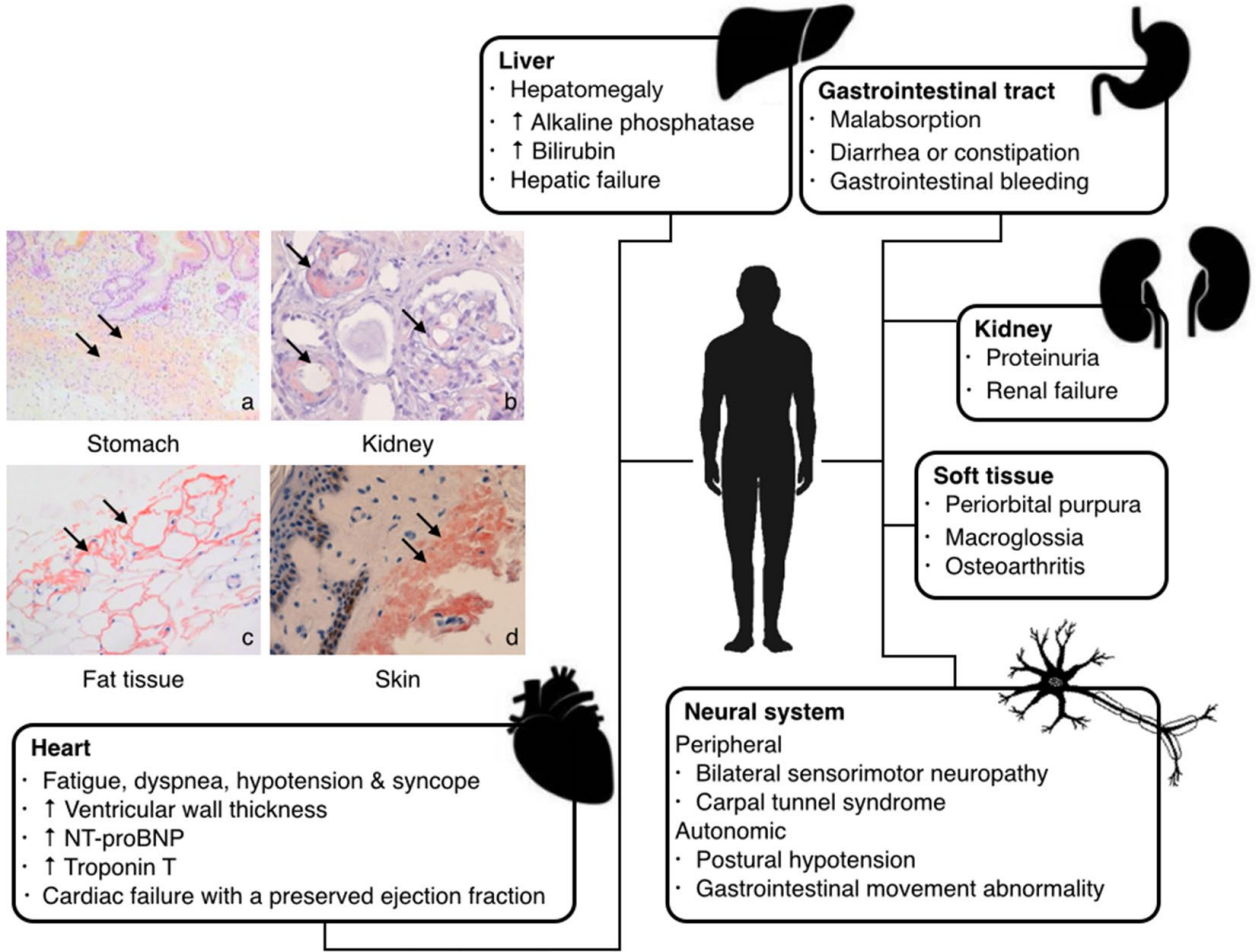
Clinical presentations and representative biopsy samples of AL amyloidosis. Free light chains secreted by the clonal plasma cells deposit in various tissues, including the heart, kidney, liver, gastrointestinal tract, soft tissues and neural systems. Detailed information about each involved organ is summarized in the figure. The representative biopsy samples of the stomach (**a**), kidney (**b**), fat tissue (**c**) and skin (**d**) stained with Congo red show amyloid deposition in these tissues, indicated with arrows (**a** was derived from Li Tian et al. [7], and **b**–**d** were from Ting Li et al. with permission [6])

Immunoglobulin light chain amyloidosis (AL) is an indolent plasma cell disorder characterized by the misfolding of free light chains (FLC) and amyloid fibril deposition in different tissues [1]. The annual incidence of this disease is around ten affected individuals per million people [2–4]. The clinical presentations of AL are various and not specific, making the disease diagnosis difficult and delay most of the time. Theoretically, any organ can be involved, except the brain, with the heart being the most commonly involved organ, followed by the kidney, liver, gastrointestinal tract, soft tissue, and peripheral and autonomic neural systems [1, 5–7] (see Fig. 1). Among these, the heart has the most significant prognostic impact [8].

Plasma cells in AL are usually indolent, but AL is regarded as a dangerous disease because only about 20% of patients could survive over 10 years, and about 25% of patients would die within the first 6 months mainly due to related organ complications [9–11].

In the past decades, significant progress has been made in understanding the molecular and genetic pathogenesis of AL. Concepts of precise treatments based on the disease’s genetic properties have emerged, which hopefully would lead to a new era of therapies in the future. This review article will focus on the pathogenesis, clinical features, and prognostic impacts of different genetic aberrations present in AL. We will also briefly discuss the influences of specific usages of germline genes and mutations on the immunoglobulin gene in amyloid formation.

## The cytogenetic landscape of AL amyloidosis

The cytogenetic landscape of AL has profound similarity with that of monoclonal gammopathy of undetermined significance (MGUS) and multiple myeloma (MM). Several studies have indicated that the cytogenetic concepts applied in MM may be transferable to AL.

MM can be classified into two distinct genetic branches: non-hyperdiploidy and hyperdiploidy based on chromosome contents, and they can further form various subclones through secondary mutations [12–14]. Similar to this, non-hyperdiploidy, which mainly involves immunoglobulin heavy chain (IgH) locus translocation, and hyperdiploidy are recognized to be the two primary clones in AL, whereas other chromosome aberrations and point mutations are frequently associated with subclone formation during disease evolution.

Patients with AL also show a high frequency of chromosome instability like other plasma cell disorders. Data shows that 60–90% of AL patients harbor at least one chromosome abnormalities and about 50–70% of them have translocations involving the IgH locus on chromosome 14 [15–17].

However, although AL shares some similar genetic features with other plasma cell dyscrasia, its distribution patterns of the cytogenetic abnormalities differ significantly from that of MGUS and MM. In AL, t(11,14) is the most common genetic abnormality with a frequency ranging from 40 to 60% [15, 18], while it only contributes to less than 20% of the cytogenetic abnormalities in MGUS and MM [12]. Meanwhile, the frequency of hyperdiploidy and secondary genetic mutations in AL seems to be lower than that of other plasma cell dyscrasia [15, 18].

Unlike other plasma cell dyscrasia, of which the prognostic roles of different genetic aberrations have been better described, less is known in AL. However, there is an increasing number of studies about this topic. Most researches about AL detected chromosome aberrations through the interphase-fluorescence in situ hybridization (iFISH) technique, using the genetic probes in MM. Different genetic aberrations involve different pathways of pathogenesis and influence prognostic outcomes and decision-making in clinical practice. The following section will focus on the basic characteristics and clinical features of the major genetic aberrations present in AL.

## Normal chromosome profile

About 10–40% of AL patients cannot be detected to harbor any chromosome aberration by the MM’s genetic probes. This patient group tends to have lower plasma cell burden, less severe cardiac involvement, longer progression-free survival (PFS) and overall survival (OS) [15–17, 19], indicating that the absence of chromosome aberration in AL may be a favorable prognostic predictor of outcome.

## Immunoglobulin heavy chain translocation

IgH translocation is the most common abnormal cytogenetic event in AL. This translocation involves the juxtaposition of different chromosomes to the IgH locus on chromosome 14, which leads to the overexpression or dysregulations of various genes, especially the cyclin gene families [12, 20–23]. In MM, t(11;14), t(4;14) and t(14;16) are the three most common IgH translocations, and t(4;14) together with t(14;16) are classified as the high-risk genetic factors [12, 24], whereas in AL, their prognostic impacts are less well defined.

### t(11;14)

Translocation t(11;14)(q13;q32) is the most prevalent genetic aberration present in AL. This translocation involves the juxtaposition of the IgH locus on chromosome 14 to the oncogene cyclin D1 (*CCND1*) on chromosome 11, leading to the overexpression of CCND1 in plasma cells [20, 25].

Translocation t(11;14) is usually genetically stable with a low rate of subclone formation and progression both in AL and non-AL plasma cell disorders [26]. Probably for this reason, t(11;14) is recognized as a neutral or favorable prognostic factor in MM [13]. However, this rule seems not to be transferable to AL. A study including 401 AL patients reported that patients harboring t(11;14) had inferior survival outcomes when their plasma cell burden was less than 10% [15]. Another study also demonstrated that the risk of death in patients with t(11;14) was 2.1 (confidence interval 1.04–6.4) [27].

Patients with t(11;14) are associated with low plasma cell clones in the bone marrow, but they tend to be loaded with a heavy FLC burden in the serum, which is shown to be related to shorter survival in several studies [28–30]. The elevated light chain level may increase the availability and tendency of the light chain precursor for amyloid fibril formation in different organs, especially the heart, which may in part explain the more unsatisfactory survival outcome in this patient group.

Reasons for the higher free light chain level in t(11;14) compared to that in non-t(11;14) remain poorly understood, but the IgH locus translocation that leads to the disruption of the IgH expression or structure may partially explain that. This is supported by the evidence from a study that identified a strong relationship between t(11;14) and the lack of an intact immunoglobulin in AL patients [31]. Data from a MM study also showed that IgH locus translocation could lead to a higher FLC ratio and higher FLC level [29]. These studies indicate that the disruption of the IgH expression or structure, probably caused by IgH translocation, can result in lower intact immunoglobulin but higher FLC level. Another article demonstrated that the elevated level of CCND1 in plasma cells was associated with more FLCs but fewer intact M-proteins, and it was an independent predictor of survival in AL patients. However, the authors did not perform the iFISH analysis in the research [32]. Since t(11;14) can lead to a high CCND1 level, whether it is the CCND1 itself, the translocation of the IgH locus, or some other reason that results in low intact immunoglobulin but high FLC production remains elusive.

The prognostic effect of t(11;14) in AL patients is still controversial, and treatment options seem to have a significant impact on the clinical outcomes of this patient group (see Table 1). Bochtler et al. first reported in 2015 that t(11;14) was associated with an inferior outcome in patients treated with bortezomib-based regimens in a study with 101 patients [33]. Further study results from other research centers also support Bochtler’s finding, indicating that patients with t(11;14) have a lower response rate and shorter median OS time when receiving bortezomib-based regimens as first-line therapy [18, 34]. However, these results should still be interpreted with caution since they are retrospective. A study also indicates that the adverse prognostic impact of t(11;14) may only restrict to the patient group with favorable factors such as younger age, less organ involvement, and Mayo stage I/II [18]. In other words, in a patient with unfavorable clinical features, the presence of t(11;14) may not have additional adverse effects.

**Table 1:** Hematologic response and survival outcome of different treatment modalities in AL amyloidosis with different genetic aberrations

Treatment modalities	Arm 1	Arm 2	Patient number (n1/n2)	Hematologic response			Survival outcome		
				End point 1	Arm 1/Arm 2	P value^c^	End point 2	Arm 1/Arm 2	P value
Bortezomib-based [34]	t(11;14)	Non-(11;14)	44/91	≥ VGPR	41%/66%	**< 0.01**	5-year OS	46%/72%	**0.026**
Bortezomib-based [33]	t(11;14)	Non-(11;14)	64/37	≥ VGPR	23%/47%	**0.02**	Median OS	8.7 months/40.7 months	**0.05**
Bortezomib-based [18]	t(11;14)	Non-(11;14)	82/89	≥ VGPR	52%/77%	**0.004**	Median OS	15.0 months/27.0 months	**0.05**
Bortezomib-based [18]	Trisomies	Non trisomies	45/125	≥ VGPR	72%/65%	0.46	Median OS	14.0 months/38.0 months	0.08
Bortezomib-based [33]	High risk aberrations^a^	Non-high risk aberrations	13/85	≥ VGPR	67%/26%	**0.008**	Median OS	NR/10.6 months	**0.04**
MD [35]	t(11;14)	Non-(11;14)	61/42	≥ VGPR	18%/22%	0.60	Median OS	38.2 months/17.5 months	0.21
MD [35]	Gain of 1q21	Non gain of 1q21	23/77	≥ VGPR	5%/25%	0.06	Median OS	12.5 months/38.2 months	**0.002**
MD [35]	Del13q14	Non del13q14	36/66	≥ VGPR	23%/18%	0.78	5-year OS	36%/33%	0.70
MD [35]	Hyperdiploidy^b^	Non-hyperdiploidy	15/84	≥ VGPR	10%/22%	1	5-year OS	40%/36%	0.70
Melphalan-based [18]	t(11;14)	Non-(11;14)	96/95	≥ VGPR	41%/54%	0.13	Median OS	23.0 months/26.0 months	0.94
Melphalan-based [18]	Trisomies	Non trisomies	49/139	≥ VGPR	39%/52%	0.2	Median OS	15.0 months/32.0 months	**0.02**
HDM + ASCT [36]	t(11;14)	Non-(11;14)	72/51	CR	41%/20%	**0.02**	Median OS	NR/93.7 months	0.07
HDM + ASCT [36]	Gain of 1q21	Non gain of 1q21	25/91	CR	22%/35%	0.32	Median OS	NR/128.8 months	0.93
HDM + ASCT [36]	Del13q14	Non del13q14	36/87	CR	21%/37%	0.13	Median OS	128.8 months/NR	0.10
HDM + ASCT [36]	Hyperdiploidy^b^	Non-hyperdiploidy	16/95	CR	27%/33%	0.77	Median OS	90.6 months/128.8 months	0.84
HDM + ASCT [36]	High risk aberrations^a^	Non high risk aberrations	9/113	CR	0%/35.2%	**0.03**	Median OS	47.4 months/NR	0.06
ASCT [18]	t(11;14)	Non-(11;14)	134/113	≥ VGPR	70%/78%	0.15	Median OS	NR/NR	0.51
ASCT [18]	Trisomies	Non trisomies	56/186	≥ VGPR	80%/71%	0.17	Median OS	NR/NR	0.98
DD [30]	t(11;14)	Non-(11;14)	53/32	Median hemEFS	24.3 months/5.5 months	**< 0.01**	Median OS	NR/19.3 months	0.07
DD [30]	Gain of 1q21	Non gain of 1q21	25/58	Median hemEFS	5.8 months/21.6 months	**0.03**	Median OS	14.8 months/NR	**0.02**
DVD [30]	t(11;14)	Non-(11;14)	23/20	Median hemEFS	19.0 months/10.0 months	0.69	Median OS	NR/NR	0.62
DVD [30]	Gain of 1q21	Non gain of 1q21	10/30	Median hemEFS	6.8 months/ 22.1 months	0.11	Median OS	9.5 months/NR	**0.02**
DRD [66]	t(11;14)	Non-(11;14)	16/15	Median hemEFS	17.3 months/22.6 months	> 0.05	–		
DRD [66]	Gain of 1q21	Non gain of 1q21	16/15	Median hemEFS	10.9 months/NR	**< 0.01**	–		
IMiD-based [18]	t(11;14)	Non-(11;14)	8/15	≥ VGPR	13%/54%	**0.04**	Median OS	12 months/32 months	**0.05**
IMiD-based [18]	Trisomies	Non trisomies	7/17	≥ VGPR	40%/38%	0.92	Median OS	17 months/23 months	0.93
Venetoclax [44]	t(11;14)	Non-(11;14)	31/11	≥ VGPR	78%/30%	**0.02**	Median OS	NR/NR	0.14

*MD* Melphalan/dexamethasone, *HDM* high-dose melphalan, *ASCT* autologous stem cell transplantation, *DD* daratumumab/dexamethasone, *DVD* daratumumab/bortezomib/dexamethasone, *IMiD* immunomodulatory drugs, *DRD* daratumumab/lenalidomide/dexamethasone, *VGPR* very good partial response, *CR* complete response, *hemEFS* hematologic event-free survival, *OS* overall survival, *PFS* progression-free survival, *NR* not reached

^a^High risk aberrations include t(4;14), t(14;16) and del17p13

^b^Using Wuilleme’s criteria.  ≥ VGPR = VGPR + CR

^c^P values ≦ 0.05 are highlighted in bold

On the other hand, in contrast to bortezomib, melphalan shows a potential to overcome the adverse effect of t(11;14). In patients receiving melphalan/dexamethasone as first-line therapy, a tendency of longer OS was observed in those with t(11;14) compared with those without t(11;14) [35]. Besides, patients with t(11;14) also benefit from high-dose melphalan (HDM) chemotherapy followed by autologous stem cell transplantation (ASCT), having a significantly higher response rate, hematological event-free survival (hemEFS) as well as a tendency of longer OS. In multivariate analysis, t(11;14) is identified as a favorable prognostic factor regarding hemEFS in HMD + ASCT therapy [36]. The mechanism behind the contradictory effects of bortezomib and melphalan-based regimens is unknown, but these findings point out the importance of carrying out the iFISH analysis to identify the underlying chromosome abnormalities and guide treatment decisions.

Selective AL patients with t(11;14) also respond well to bcl-2 inhibitor, venetoclax. Bcl-2 is a protein that regulates the programmed cell death pathway in normal cells. Cells overloaded with bcl-2 fail to undergo apoptosis but accumulate in the body. Plasma cell disorders, including MM and AL, especially those with t(11;14), are associated with the overexpression of bcl-2 [37–39]. The first case report about the efficacy of venetoclax on AL comes from Leung et al., demonstrating that venetoclax could induce a complete response in a patient with t(11;14) plateaued on the standard bortezomib + cyclophosphamide + dexamethasone regimen [40]. Further findings also confirm that the addition of venetoclax could lead to deeper hematological and organ response in a relatively short period [41–44], even in patients without t(11;14) [42]. A recent retrospective study involving 43 patients with relapsed/refractory AL demonstrates that venetoclax can achieve a VGPR/CR rate of 63% in total, and the presence of t(11;14) predicts longer PFS compared to non-t(11;14) [44]. However, attention should be paid to the increased risk of infection in venetoclax-based therapies, and the usage of prophylactic antibiotics and intravenous immunoglobulin is recommended [42, 44].

Daratumumab, a monoclonal CD38 antibody, has also shown promise outcome in t(11;14)-positive patients as a salvage therapeutic modality, demonstrating a longer hemEFS [30]. This may be explained by the higher CD38 expression level in patients with t(11;14) [45], which can promote the antibody-dependent cytotoxicity effect of daratumumab [46].

In summary, t(11;14) is associated with an increased FLC level in AL and poorer survival outcomes under bortezomib-based regimens, especially in low-risk patients. Melphalan and novel agents, including venetoclax and daratumumab, may be capable of overcoming the adverse effect of t(11;14) in AL treatments.

### t(4;14)

Translocation t(4;14)(p16.3;q32) results in the dysregulation of two genes on chromosome 4, the fibroblast growth factor receptor 3 (*FGFR3*) and multiple myeloma SET domain (*MMSET*), together with the formation of IGH/MMSET hybrid transcripts [47–49]. This translocation contributes to about 20% of the genetic abnormalities in MM and is regarded as a poor prognostic factor [50]. Different from MM, t(4;14) comprises only about 2% of chromosome abnormalities of AL detected by iFISH method [15], but a rate of 14% (6/42) was reported in a study using the reverse transcriptase-polymerase chain reaction to detect the IGH/MMSET transcripts [51]. Due to the rare entity of the AL itself and the low prevalence of t(4;14), data regarding the clinical feature and prognostic effect of t(4;14) in AL are scarce. In bortezomib-based regimens, no adverse prognosis effect was detected in t(4;14), suggesting that bortezomib-based regimens should be considered in this patient group as in MM [13]. Another study observing the effect of high-risk cytogenetic aberrations including t(4;14), t(14;16) and deletion of 17p13 in ASCT indicates that these aberrations may confer an unfavorable prognosis [36]. However, the sample sizes in these studies are small, and large-scale studies are needed to confirm these findings.

### t(14;16)

Translocation t(14;16)(q32;q23) has been described in about 2% of AL, a frequency slightly lower than that of MM (~ 5%) [12, 27]. This translocation leads to the rearrangement of the *MAF* gene, which encodes a transcription factor binding to *CCND2* [13]. A close relationship between increased FLC and IgH translocation, especially t(14;16) is observed in a MM study [29]. Whether AL patients with t(14;16) have an elevated FLC level and an increased predisposition to amyloid fibril formation remains poorly understood. Two studies conducted by Bochtler et al. detected an adverse impact of t(14;16) on AL patients undergoing ASCT with melphalan but not on patients receiving bortezomib-based regimens [33, 36], but the patients numbers were small to reach a definitive conclusion.

### Other IgH locus translocations

Other IgH locus translocations, such as t(14;20) and t(6;14), are rarely found in AL [15, 18, 52]. T(14;20) was reported to be associated with MAF-B expression in AL cell lines derived from a patient that eventually evolved into MM [52]. For t(6;14), this may give rise to *CCND3* dysregulation [13, 53]. In MM, t(14;20) is regarded as a high-risk factor while t(6;14) is a standard-risk factor [13, 54]. However, there is still no evidence to support their prognostic roles in AL.

## Hyperdiploidy

Hyperdiploidy is a well-recognized alternative pathogenetic pathway in plasma cell disorders. It is characterized by the presence of extra copies of multiple chromosomes, especially odd-numbered chromosome 3, 5, 7, 9, 11, 15, 19 and 21 [12, 55].

Because of the low percentage and low proliferative rate of plasma cells, conventional karyotypic analysis methods are not suitable for the study of ploidy in AL [56, 57]. To overcome this problem, Wuilleme et al. established a definition of hyperdiploidy based on iFISH, which requires trisomies of at least 2 of 3 chromosome combinations to define hyperdiploidy [57]. According to this definition, about 30% and 50% of MGUS and MM respectively have hyperdiploidy, while the frequency of hyperdiploidy in AL is only about 10% [11, 12, 26, 31, 58]. Hyperdiploidy is associated with intact immunoglobulin, κ light chain restriction, older age, more plasma cell clones, lower CD38 expression level, and higher subclone formation frequency [26, 45, 58]. The prognostic effect of hyperdiploidy seems to be neutral in patients undergoing standard or high-dose melphalan therapies [35, 36], but an adverse outcome was detected in a study observing the effect of daratumumab-based therapy [30].

The application of Wuilleme’s definition of hyperdiploidy will inevitably underestimate the prevalence of this genetic aberration. A study from Granzow et al. using high-density copy number arrays detected 25% (29/118) of patients as hyperdiploidy, including six patients not identified as hyperdiploidy by Wuilleme’s criteria [59]. Another study conducted by Ozga et al. described a broader definition of hyperdiploidy that included gains/trisomies of 2 or more of any chromosome loci. In this study, 38% of patients harbored hyperdiploidy, which was confirmed to be associated with shorter PFS and OS in the multivariate analysis [17].

Overall, hyperdiploidy seems to confer a neutral or inferior outcome in AL patients, which is contradictory to results from MM studies that indicate a favorable prognostic effect of hyperdiploidy [12, 13, 60]. The transferability of the classically defined hyperdiploidy in the iFISH method, previously used in MM, to AL remains uncertain, and further studies are needed to define and validate the criteria of hyperdiploidy in AL based on the iFISH method.

### Trisomies

Trisomies, defined as the presence of extra copies of one or more chromosomes, include both hyperdiploidy and mono-chromosome gains. 20–30% of AL patients harbor trisomies, with trisomies 9 being the most common genetic aberration, followed by trisomies 19, 15, 5, 18, 11, 7, 3, 17, 4, 21 and 14 [15, 18, 59]. High dFLC (involved FLC minus uninvolved FLC) and bone marrow plasmacytosis are associated with the presence of trisomies [15]. In general, trisomies predict a worse outcome, especially in patients with plasma cell burden greater than 10% [11, 15]. This is different from the prognostic impact of trisomies in MM, where trisomies seem to attenuate prognostic risk [61, 62].

Reasons for the adverse impact of trisomies remain unknown. One of the possible reasons is that trisomies correlate with a higher clone evolution rate, indicating a more malignant potential of this disease. Moreover, the high dFLC level and plasma cell burden in patients with trisomies may lead to more amyloid fibril formation in targeted tissues and worsen organ functions. Further studies are still needed to explore the role of trisomies in AL. Besides, it might be necessary to subdivide trisomies into hyperdiploidy group, usually regarded as the primary cytogenetic event, and mono-chromosome gain group, regarded as the secondary cytogenetic event, as the underlying mechanisms of these two groups differ and they may have different prognostic impacts.

## Secondary genetic events

Malignant plasma cells of an individual AL patient are not usually genetically identical, but harbor some additional aberrations, forming various subclones. This intraclonal heterogeneity in AL is the consequence of secondary cytogenetic events occurring during disease progression. Studies about AL’s subclone architecture reveal that gain of 1q21, deletion of chromosome 13/13q, 17p and 8p21, secondary IgH translocations, and point mutations are frequent progression-related aberrations [26]. Hyperdiploidy is more often associated with subclone formation, but t(11;14) is less often [26]. Secondary genetic aberrations could induce additional activations of oncogenes and inactivations of tumor suppressor genes, promoting disease progression. Under the selective pressure of treatments, the outgrowth of drug-resistant clones may arise when intraclonal heterogeneity is present [63, 64]. Hence, in general, the presence of secondary cytogenetic events in AL confers an unfavorable survival outcome.

### Gain of 1q21

In AL, 1q21 gain usually results from the gain of the long arm of chromosome 1, rather than trisomies 1 [59]. 20–30% of AL patients harbor this genetic aberration, similar to the rate in MM [15, 35, 59]. However, in contrast to MM, in which the gain of 1q and loss of 1p always occur together [12, 13], deletion of chromosome 1p is rarely found in AL. The presence of 1q21 gain is associated with plasmacytosis [35, 59], a tendency of progression to MM [31], a greater risk of cardiac involvement [65], lower CD38 expression level, and an inferior or neutral survival outcome [45]. Bochtler et al. also showed a close relationship between the gain of 1q21 and hyperdiploidy [58], but Granzow et al. implied that this might only be true when Wuilleme’s definition of hyperdiploidy was used [59].

Probably, due to the low CD38 level, patients with 1q21 gain achieved less satisfactory outcomes than those without this genetic aberration when daratumumab-containing regimens were adopted [30, 66]. One study investigating the impact of 1q21 on the standard melphalan/dexamethasone treatment also demonstrates that 1q21 is an independent adverse prognostic factor for survival [35]. However, no significant prognostic impact of 1q21 has been identified in AL patients receiving bortezomib-based regimens or HDM + ASCT [33, 36].

The responsible genes underlying this chromosome aberration are unknown. The amplification of cyclin kinase subunit 1B (CKS1B) has been identified in a significant proportion of MM patients with the gain of 1q21 and is associated with an unfavorable survival outcome [67, 68], but data regarding the role of CKS1B in AL is laking. Granzow et al. reported a higher rate of concomitant loss of chromosome 14q (33% vs. 12%, p < 0.005) and 16q (24% vs. 1%, p < 0.001) in patients with the gain of 1q21 compared to those without this genetic aberration [59]. They suggested that two tumor suppressor genes *IDP2* and *MLH3,* located in the minimal common region of deletions on chromosome 14q, may be responsible for the disease pathogenesis, but there is no evidence by now to validate this notion.

### Monosomy 13/deletion of 13q

Chromosome 13 abnormalities, including monosomy 13 and deletion of 13q (del13q), can be detected in about one-third of patients with AL [15, 18, 25, 59] and a half with MM [69, 70]. The presence of del13q in MGUS is associated with a higher risk of progression to MM, while its prognostic effect in MM tends to be neutral [53]. In AL, monosomy 13/del 13q are the only chromosome abnormalities identified so far to have cardiac tropism and are associated with high NT-proBNP level [15, 17, 65]. They also correlate with plasmacytosis and a high dFLC level [15, 17]. However, no prognostic significance has been identified related to these genetic aberrations. Treatment modalities, including standard dose melphalan, HDM + HSCT, bortezomib-based, and daratumumab-based therapies, seem not to influence the survival outcome [30, 33, 35, 36]. Haploinsufficiency of *Rb1* gene, located on chromosome 13q, has been proposed to be responsible for disease progression in MM [12, 71]. A similar effect may be present in AL and needs further exploration.

### Deletion of 17p

Deletion of 17p (del17p), specifically at the locus of 17p13, occurs at a rate of no more than 5% in AL [15, 26]. Probably due to its rareness, no significant prognostic impact of this genetic aberration has been reported to date in AL [33, 36]. The largest-scale study about this genetic aberration in AL comes from a collaboration of seven countries involving 44 patients with del17p [72]. In this study, 95% of patients had cardiac involvement, and the median plasma cell burden was 22%. Half of the patients could achieve a VGPR or CR, with two-thirds of them receiving bortezomib-based therapies. The median OS for this patient population was 49 months, which seems to be comparable to the general survival outcome of AL patients [10]. However, this study suggested that a higher burden of del17p may predict inferior survival outcomes because patients with > 50% del17p tended to live shorter than those with ≤ 50% del17p (median OS: 28 months vs. 52 months, p = 0.08).

Research about the pathogenetic effect of del17p in AL is scarce. Del17p can result in the deletion of an important tumor suppressor gene, *p53*, the dysfunction of which leads to disease progression of various tumor kinds, including plasma cell disorders [73–75]. In MM, del17p is regarded as a strong adverse predictor of survival and is associated with a disposition to develop extramedullary disease [12, 13]. Several studies have demonstrated that the inactivation of p53 in MM may promote plasma cells to survive at extramedullary locations without undergoing apoptosis [76, 77]. A similar effect of the inactivation of p53 in AL may also occur, as two AL cell lines generated from a single person also have p53 loss, indicating that the absence of p53 may allow plasma cells to proliferate independently of the bone marrow environment [52].

### Other chromosome aberrations and driver mutations

Deletion of 8p21 and secondary IgH translocation occur at a low frequency in AL, so their clinical characteristics and prognostic effects are poorly understood [26, 31, 59], which highlights the need for multi-center cooperations to investigate specific genetic abnormalities of this rare disease. Trisomies 9 and 19 are the two most common chromosome gains in AL. A study involving 27 patients indicates that both of them are associated with an inferior PFS, but larger-scale studies are needed to confirm the result [65].

Whole exome sequencing (WES) can detect specific point mutations undetectable by the conventional iFISH method. A series of recurrent mutations in AL have been identified, but they all occur at a very low rate and there is no consistent result regarding these recurrent mutations in recent studies [65, 78–80]. A research based on WES and targeted gene sequencing reported that three recurrent mutations on *ASB15*, *ASCC3* and *HIST1H1E* are associated with inferior OS [80], but other studies were unable to identify any linkage between genetic mutations and patients’ outcomes [65, 78, 79]. In terms of MM related driver genes in AL, they are present at a frequency similar to that in MGUS, which is lower than in MM [65, 79]. *KRAS* and *NRAS* activated mutations, commonly found in MM, are only identified in a few cases in AL [65, 78, 79], but one study identified mutation on *KRAS* as a recurrent mutation without survival significance [80].

## Genomic alterations related to amyloid formation

The cytogenetic landscape of AL displays profound similarity with that of MGUS and MM, confirming the concept that they are related plasma cell dyscrasia. However, these findings are insufficient to explain why plasma cells in AL are more prone to secret amyloid-generating proteins. Fortunately, research on the immunoglobulin light chains (IgL) helps shed some light on the pathogenesis of this disease. Data in the previous studies indicate that organ tropism in AL may be the function of the usage of specific IgL germline genes [81–88] (see Table 2). For example, AL patients with κ light chain have more liver involvement [28, 87, 89, 90]. On the other hand, the germline genes *IGLV6-57* and *IGLV1-44* are linked to increased renal and cardiac involvement, respectively [81, 82, 84, 86–88]. Besides, there is an overrepresentation of specific germline genes in AL, that is, some germline genes, such as *IGLV6-57, IGLV3-01* and *IGLV2-14*, occur at a higher frequency in AL than other plasma cell dyscrasia or normal B cells [81, 87, 88, 91].

**Table 2:** Organ tropism of AL amyloidosis related to IgL germline genes

Light chain germline gene	Prevalence in AL (%) [28, 87, 88]	Organ tropism	Other associated features
κ light chain	~ 20	**↑ Hepatic involvement** [28, 79, 87, 90]	Better survival outcome after ASCT [90]
		↑ Soft tissue involvement [85]	
		↑ Bone involvement [85]	
*IGKV1-05*	2	May ↓ renal involvement [87]	Inferior overall survival [87]
		May ↑ advanced cardiac disease [87]	
*IGKV1-33*	5	↑ Hepatic involvement [87]	High circulating dFLC [87]
		May ↓ peripheral nerve involvement [87]	
*IGKV3-20*	2	Localized AL amyloidosis [87]	–
IGKV4-01	5	↑ Gastrointestinal involvement [88]	
		↑ Soft tissue involvement [88]	
λ light chain	~ 80		
*IGLV1-44*	6	**↑ Cardiac involvement** [81, 82, 86, 87]	↓ Rate of trisomies [87]
		May ↑ multisystem involvement [81, 82]	
		↓ Hepatic involvement [88]	
IGLV2-08	3	**↑** Lung involvement [88]	Associated with IgM light chain amyloidosis [88]
*IGLV2-14*	9	↑ Gastrointestinal localized AL [87]	**↑** Intact immunoglobulin [87]
		May ↑ cardiac involvement [82]	↓ Circulating dFLC [87, 88]
		May ↑ peripheral nerve involvement [87], but another study did not support this finding [88]	Associated with IgM light chain amyloidosis [88]
		May ↑ multisystem involvement [82]	
*IGLV3-01*	9	May ↑ cardiac involvement [82]	–
		May ↑ multisystem involvement [82, 84]	
		May ↓ advaced cardiac disease [86, 87]	
		May ↓ renal involvement [87]	
*IGLV3-19*	2	May ↑ cardiac involvement [87]	
*IGLV3-21*	5	↓ Renal involvement [87]	**↑** Rate of monosomy 13q [87]
*IGLV6-57*	13	**↑ Renal involvement** [81, 82, 84, 87, 88]	**↑** Rate of t(11;14) [87]
		May ↓ cardiac involvement [14, 86]	↓ Rate of trisomies [87]
			Better survival outcome after ASCT [81]

The contents highlighted in bold indicate that they are supported by 2 or more evidences with statistical significance

Somatic mutations in the IgL genes, generated in the process of immunoglobulin synthesis, add complexity to the IgL structure. The burden of the somatic mutations and the specific site that a mutation occurs can influence the stability and amyloidogenicity of IgL [91–95]. A model from a recent study based on somatic mutations in IgL displays high sensitivity and specificity to predict the free light chain toxicity [96]. Moreover, the introduction of specific point mutations in the IgL locus could attenuate the free light chain toxicity [96–98], which underlines the possibility of gene therapy in AL.

**Fig. 2:**
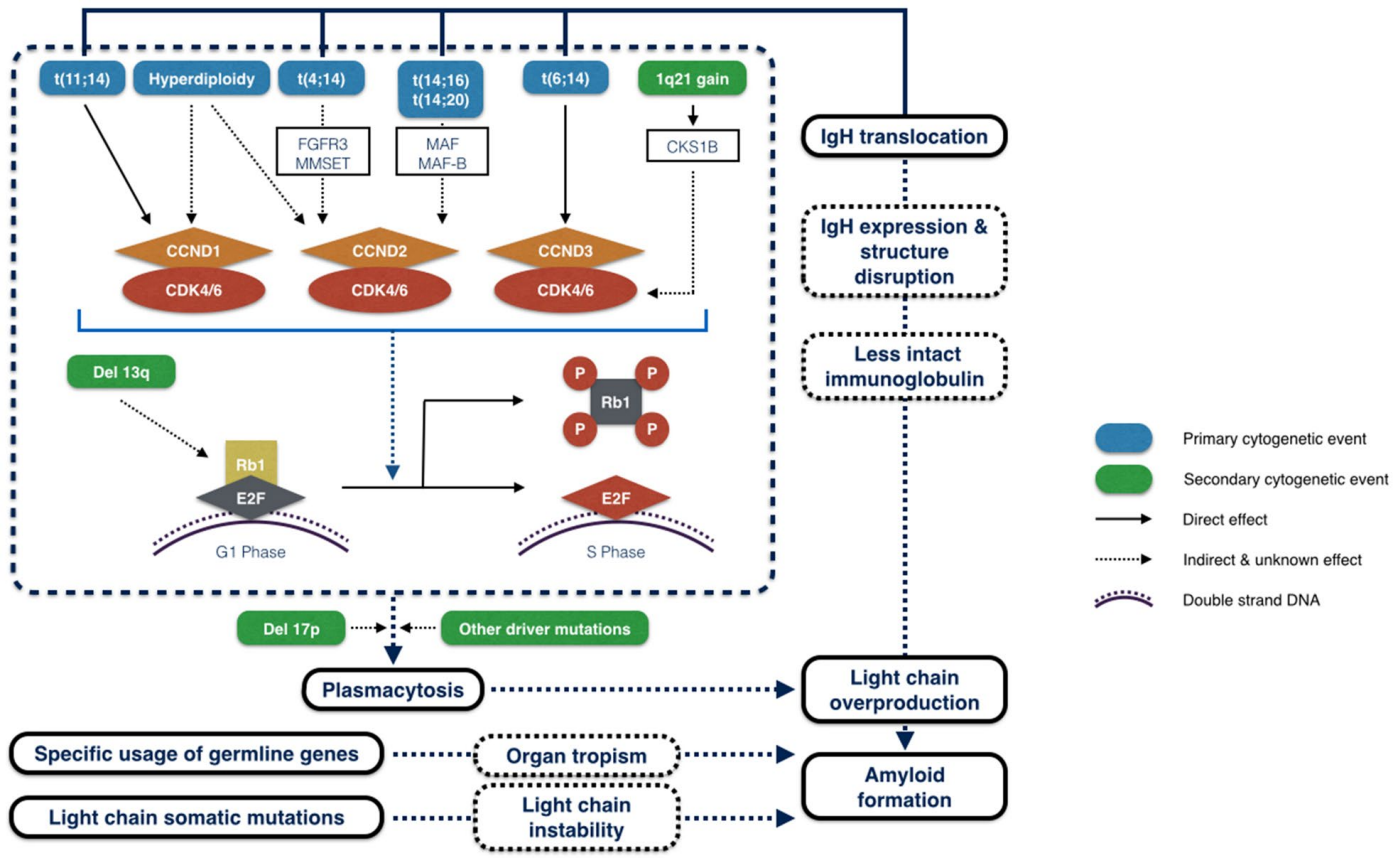
Amyloid formation of AL amyloidosis in a genetic base. Primary cytogenetic aberrations, including IgH translocation and hyperdiploidy, upregulate the cyclin D (*CCND*) gene family, which then forms complexes with cyclin-dependent kinase 4 (CDK4) and CDK6. As a consequence, the retinoblastoma protein 1 (Rb1) is phosphorylated, releasing transcription factor E2F. This transcription factor initiates the transcription of genes necessary for G1-to-S phase transition and results in plasma cell proliferation. Secondary cytogenetic events, including 1q21 gain and loss of 13q, can exacerbate the uncontrolled cell proliferation by promoting CCND-CDK activity and impairing Rb1 function, respectively. Deletion of 17p and other driver mutations also promote plasmacytosis through other pathways. This large number of plasma cells produce excess light chains that then deposit in different tissues. Meanwhile, IgH translocation may disrupt the IgH expression or structure and lead to low intact immunoglobulin but high free light chain level. Moreover, the specific usages of immunoglobulin light chain (IgL) germline genes facilitate amyloid deposition in specific organs, and somatic mutations in IgL can produce unstable light chains that are more likely to form fibrils. CKS1B: cyclin kinase subunit 1B; MMSET: multiple myeloma SET domain; FGFR3: fibroblast growth-factor receptor 3; MAF, MAF-B & E2F: transcription factor; P: phosphorylation

Altogether, these findings indicate that the formation of amyloid fibril in various organs is a multifactorial process involving chromosome aberrations, secondary mutations, specific usage of IgL germline genes, IgL somatic mutations, and some other unidentified reasons (see Fig. 2).

## Conclusions

AL amyloidosis is a plasma cell disorder having distinct features from that of MM, although they indeed share some genetic similarities. Each genetic aberration in AL involves distinct pathogenetic pathways and displays some special clinical features. However, these are insufficient to explain the most significant feature of AL, the amyloid formation. Studies about the IgL indicate that specific usages of IgL germline genes and point mutations in the IgL locus may be capable of predicting the organ tropism and fibril formation tendency of this disease. Treatment options based on the cytogenetic features of AL and novel therapies targeting amyloid formation have a promising future. To date, due to the low incidence of AL, most studies of this disease are retrospective. Therefore, multi-center cooperations are needed to investigate specific cytogenetic abnormalities and conduct sizable clinical trials in this disease. The development of the predictive model, based on the cytogenetic properties of AL, may help improve the early diagnosis and clinical outcomes in AL patients.

## Data Availability

Not applicable.

## Abbreviations

AL: Immunoglobulin light chain amyloidosis
FLC: Free immunoglobulin light chain
MGUS: Monoclonal gammopathy of undetermined significance
MM: Multiple myeloma
IgH: Immunoglobulin heavy chain
iFISH: Interphase-fluorescence in situ hybridization
PFS: Progression-free survival
OS: Overall survival
CCND1: Cyclin D1
HDM: High-dose melphalan
ASCT: Autologous stem cell transplantation
hemEFS: Hematological event-free survival
FGFR3: Fibroblast growth factor receptor 3
MMSET: Multiple myeloma SET domain
CKS1B: Cyclin kinase subunit 1B
WES: Whole exome sequencing
IgL: Immunoglobulin light chain
